# Energetic microdomains and the vascular control of neuronal and muscle excitability: Toward a unified model

**DOI:** 10.1113/JP289492

**Published:** 2026-01-29

**Authors:** L. Fernando Santana, Scott Earley

**Affiliations:** 1 Department of Physiology & Membrane Biology, School of Medicine, University of California, Davis, California, USA; 2 Department of Pharmacology and Physiology, University of Rochester Medical Center, Rochester, New York, USA

**Keywords:** ATP, bioenergetics, calcium, metabolism, microcirculation, mitochondria, neuromuscular coupling

## Abstract

Ion channels are often depicted as autonomous membrane switches, yet their function depends on upstream vascular and mitochondrial processes that deliver ATP to maintain the ionic gradients essential for cellular excitability. Here, we propose a unifying framework, in the form of a capillary–mitochondria–ion channel (CMIC) axis, that links microvascular architecture to beat-to-beat performance in the heart and spike-to-spike behaviour in the brain. In this formulation, capillaries define the spatial resolution of oxygen and energy substrate delivery, while mitochondria serve as the critical intermediary that couples vascular supply to ion channel performance. CMIC coupling is critical in both cardiac function, where each cycle initiates with an electrical spike in pacemaking cells, and neural activity, where electrical spikes encode language, memories and other cognitive processes. Both neurons and cardiomyocytes have limited metabolic reserves, making them vulnerable to microvascular changes. Accordingly, these shared energetic demands, the brain and heart, exhibit similar microvascular topologies where capillary density scales with local metabolic demand. The myocardium is far more densely vascularized than the cerebral cortex, consistent with the higher energetic cost of pacemaking and contraction relative to individual neuronal spikes. Within this axis, mitochondria shape ATP waveforms to power rapid ionic gradients and Ca^2+^ cycling. Changes in any CMIC component, such as capillary rarefaction or mitochondrial dysfunction, alter energetics and thus cellular excitability, with effects ranging from adaptive to pathological depending on severity. Recognizing excitability as a product of vascular-initiated processes shifts the therapeutic focus toward preserving microvasculature function, retuning mitochondria–channel coupling and restoring capillary signalling.

## Graphical Abstract

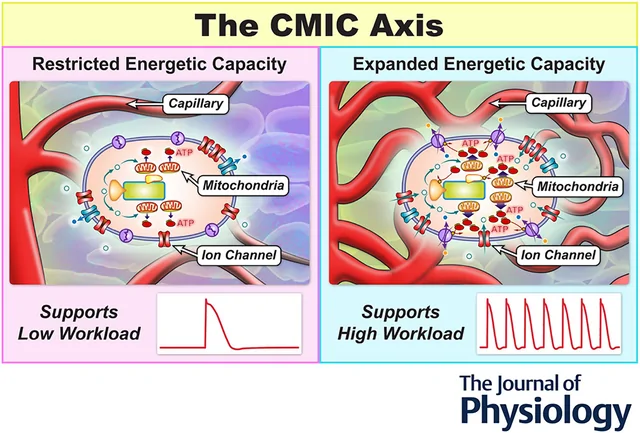

The capillary–mitochondria–ion channel (CMIC) axis scales structural resources to match functional workload. (Left) In settings of restricted energetic capacity (e.g. cortical neurons), sparse capillary networks and modest mitochondrial pools set a lower energetic ceiling, sufficient to support phasic, low-workload excitability. (Right) In settings of expanded energetic capacity (e.g. cardiac pacemakers), a dense capillary lattice and increased mitochondrial volume extend the metabolic bandwidth. This high-capacity supply chain generates the rapid, beat-to-beat ATP fluxes required to sustain high-workload, rhythmic firing. The CMIC framework demonstrates that excitability is an emergent property constrained by the density of the vascular–metabolic infrastructure.

‘Diligence is often rewarded with discovery. It is simply a matter of applying a recent technique to a problem that has lain dormant for some time.’

(Ramón y Cajal, 1897)

‘The tragedies in life are largely arterial’

Sir William Osler (cited in Criado, 2011)

## Introduction – reframing excitability

Across the heart and brain, action potentials (APs) drive function. In the cardiac setting, an AP initiates a cascade – Ca^2+^ entry via L-type Ca^2+^ channels, Ca^2+^-induced Ca^2+^ release from the sarcoplasmic reticulum (SR) and a cell-wide rise in intracellular Ca^2+^ that induces excitation–contraction coupling, causing a single beat of the heart (Bers, 2001; Hille, 2001; Hodgkin & Huxley, 1952; Rieke, 1997). In neurons of the brain, a series of APs, in the form of spike trains or bursts, encode information.

The energetic cost of an AP and the events activated by each spike are not equivalent across organs (Poole & Musch, 2023). Neurons, characterized by brief (~2–4 ms) APs, spend most of their ATP budget on restoring ion gradients and supporting synaptic transmission (Rangaraju et al., 2014; Trevisiol et al., 2017). In the heart, a single, much longer AP (~50 ms in mouse; 200–300 ms in human) triggers contraction, with myosin ATPase and the sarcoplasmic/endoplasmic reticulum Ca^2+^ ATPase (SERCA) pump dominating consumption, substantially increasing the per-event cost (Attwell & Laughlin, 2001; Howarth et al., 2012; Rhana et al., 2024, 2025).

Ion channels are often portrayed as gatekeepers whose behaviour is set by voltage, ligands and intrinsic kinetics. This channel-centric view misses a crucial constraint: excitable cells live close to energetic limits. Channels not only sense voltage, intracellular Ca^2+^ or ligands: they also operate within local metabolic microdomains in which energy waxes and wanes with blood flow and oxidative ATP supply.

In this review, synthesizing evidence from cardiac and neurovascular systems, we propose that cellular excitability emerges from the integrated function of a tripartite system comprising capillaries, mitochondria and ion channels – designated as the capillary–mitochondria–ion channel (CMIC) axis. Within the CMIC framework, local vascular architecture and activity-synchronized ATP dynamics determine ion channel function and cellular excitability. Specifically, capillaries establish the upper limit of substrate delivery; mitochondria determine the temporal dynamics of ATP production; and Ca^2+^-mediated signalling coordinates oxidative phosphorylation timing, thereby regulating channels directly gated by ATP, such as ATP-sensitive K^+^ K_ATP_ channels, and ATP-dependent pumps (including SERCA and Na^+^/K^+^ ATPase).

The novelty of this conceptualization lies in its explicitly multi-scale *in situ* integration – from capillary geometry and blood-flow dynamics to moment-by-moment channel gating. This framing repositions channels from isolated actors to residents of energy-limited microdomains, where metabolic context tunes excitability in real time. In this context, ‘energetic microdomain’ refers to a local neighbourhood in which capillary delivery, mitochondrial ATP production and ATP consumption by channels/pumps are tightly co-registered in space and time. A companion concept in this reframing is ‘bioenergetic noise’, which denotes beat-to-beat (or burst-to-burst) fluctuations in ATP availability that perturb channel kinetics and pump performance. CMIC extends the classical view of the neurovascular unit and excitation–metabolism relationships by explicitly linking capillary geometry/topology to moment-by-moment mitochondrial ATP waveforms and, in turn, channel gating, ion transport, vesicle recycling and contraction.

Excitable cells such as cardiac myocytes (Rhana et al., 2024, 2025) and neurons (Rangaraju et al., 2014) function close to their energetic limits. Nowhere is this more evident than in the heart. The human heart beats ~100,000 times per day, and each beat – arising from a surge in ion pumping, Ca^2+^ cycling and actin–myosin cross-bridge formation – is a major energetic drain that consumes a vast amount of ATP. Yet recent real-time measurements of intracellular ATP in beating cardiomyocytes challenge the implicit assumption of abundant energy reserves (Eisner & Murphy, 2024; Murphy & Steenbergen, 2008). Instead, these experiments reveal rapid, beat-to-beat oscillations in ATP levels, with diastolic [ATP]_i_ dropping below 1 mM – an order of magnitude lower than classical estimates (Rhana et al., 2024).

In other words, by the end of each beat, the cardiac myocyte is operating at relatively low ATP, which can alter multiple processes. A fall from ~0.5 to 0.1–0.2 mM ATP – together with rises in ADP/P_i_ – slows SERCA and the Na^+^/K^+^-ATPase (via reduced Mg-ATP availability and product inhibition), leading to slower Ca^2+^ reuptake, broader/longer Ca^2+^ transients, and lower SR Ca^2+^ load; the diminished dyadic Ca^2+^ signal blunts Ca_V_1.2-dependent inactivation, alters Na^+^/Ca^2+^ exchanger drive and could even permit activation of K_ATP_ channels that are usually electrically silent when energy is ample (Noma, 1983). This implies that transient metabolic dips and surges that accompany each heartbeat could have a broad impact on excitability.

Thus, far from having a large ATP safety margin, cardiac myocytes must refill their ATP between every beat, a process that relies on tight coupling of Ca^2+^ signals to mitochondrial energy production to boost output on demand. The importance of this synchronization of mitochondrial respiration with beat-to-beat demands is supported by the observation that downregulation of mitofusin-2 (Mfn2), a key tethering protein that connects mitochondria to SR Ca^2+^-release sites, blunts the amplitude of these beat-synchronized ATP transients (Chen et al., 2012; Dorn et al., 2015). Such findings cast the cardiac AP in a new light; instead of simply an electrical event, it is an electro-metabolic ‘handshake’, in which each beat’s ion fluxes are ‘paid for’ in real time by localized ATP production.

Neurons, too, have limited long-term energy reserves and are highly dependent on a continuous supply of blood. In the brain, this continued delivery is achieved through two mechanisms: autoregulation, which maintains blood flow in the face of blood pressure fluctuations, and neurovascular coupling, a rapid, feedback-driven mechanism that increases local blood flow (functional hyperaemia) to regions of enhanced neuronal activity. Recent work has unveiled a new protagonist in this story: the brain’s extensive capillary network (Longden et al., 2017; Thakore et al., 2021).

No longer considered passive conduits, capillaries are now known to be real-time sensors of neural activity and intermediaries between this activity and blood delivery. Recent groundbreaking studies from the Nelson (Longden et al., 2017) and Earley (Thakore et al., 2021) labs have shown that brain capillary endothelial cells (ECs) detect byproducts of neuronal firing, such as extracellular K^+^ and reactive oxygen species (ROS) metabolites, and translate this information into signals that propagate along the capillary network and reach upstream arteries and arterioles, where they cause a dilatory response and an increase in local blood flow – and thus delivery of oxygen and glucose – to neurons. Such capillary-initiated signalling embodies the concept of energetic microdomains: namely, that a neuron’s excitability is sustained by the reaction of a microvascular element in its immediate vicinity. Without this on-demand hyperaemic response, active neurons would quickly deplete their ATP stores – akin to a failing heart – producing a state in which channel activity, ion gradients and synaptic fidelity are compromised. Notably, mitochondria in neurons are shuttled along axons and dendrites to sites of high activity, pausing at synapses to generate local ATP and buffer Ca^2+^ and thereby reinforce these energetic microdomains (Schwarz, 2013; Sheng & Cai, 2012; Wang & Schwarz, 2009). The positioning, and even fusion–fission dynamics, of neuronal mitochondria ensure that bursts of activity are met with equally dynamic ATP support, preventing neural circuits from experiencing an energy deficit.

The concept of localized energetic constraints is supported by recent high-resolution mapping of cerebral oxygenation in awake animals. Rather than a uniform ‘sea’ of oxygen, the cortical landscape is punctuated by spontaneous, transient ‘hypoxic pockets’ – discrete microvolumes where tissue $P_{{\text{O}}_{2}}$ drops significantly below the surrounding average (Beinlich et al., 2024; Moeini et al., 2018). These events, which are exacerbated by ageing and capillary flow heterogeneity, occur even in the absence of occlusion (Moeini et al., 2018). In the CMIC framework, these pockets represent areas where the local safety margin is vanishingly thin, making the associated neuronal circuits uniquely vulnerable to capillary stalling or mitochondrial dysfunction. Their existence confirms that aerobic capacity is not a fixed constant but a dynamic variable defined by the immediate microvascular architecture (Beinlich et al., 2024).

The common theme emerging from cardiac and neural physiology is that of a precarious, but highly coordinated, balancing act between excitation and metabolism. In both tissues, the local vascular geometry (i.e. the distribution of capillaries and its impact on how swiftly blood can be delivered) and mitochondrial responsiveness (where and how quickly ATP is produced) together govern ion channel performance. A cardiac myocyte living off pulsatile coronary perfusion and a cortical neuron relying on activity-gated cerebral blood flow may seem worlds apart, yet they operate under strikingly analogous constraints: both are one burst of activity away from ATP bankruptcy, and both have evolved convergent, just-in-time mechanisms to avert it. We propose that the CMIC axis is the unifying lens through which to view and understand this tight interplay. According to this framework, excitability is fundamentally a multi-scale phenomenon in which the fidelity of ion channel function (and thus APs and spikes) is governed by integrated events ranging from capillary-mediated arteriole dilation and red blood cell (RBC) movements to mitochondrial Ca^2+^ transients and nanodomains of ATP around channel complexes. Through this CMIC axis, heartbeats and brain activity share a common currency and a common tension, namely the need to continuously generate and consume ATP in synchrony with their activity.

In the sections that follow, we develop this concept of system-embedded excitability. We review recent data from cardiac muscle, including rhythmic intracellular ATP transients and their impact on membrane currents, together with insights from neurovascular coupling and ATP dynamics in the brain, such as capillary-initiated electrical and Ca^2+^ propagating signals, to build a new integrated model of ion channel regulation. By reframing ion channels as end-stage participants in an energy supply chain rather than disinterested spectators, we aim to crystalize a bold vision for excitability research. The ‘capillary-to-channel’ perspective compels modification of traditional views, reframing ion channel behaviour as an emergent feature of the cell’s energetic system rather than an insulated, intrinsic function. Ultimately, appreciating this intimate linkage of vascular supply, mitochondrial power and membrane excitability could unify our understanding of cardiac and neural function and illuminate novel strategies for protecting these excitable tissues when they are running on their last drops of fuel.

## Capillary networks – the spatial foundation of energetics

The viability of an excitable cell is fundamentally determined by the structural configuration of its metabolic supply pathways. Capillaries function not as passive conduits but as an integrated sensory network, with each vessel serving as a discrete unit in a comprehensive metabolic surveillance system that monitors activity at every sarcomere and synapse to regulate substrate delivery accordingly.

### Geometric limits of diffusion.

Theoretical models of capillary-mediated oxygen transport have framed our understanding of tissue perfusion ever since Krogh’s (1919) pioneering work on skeletal muscle at the turn of the last century. This model assumed that each capillary supplies oxygen to a surrounding cylindrical tissue region and further assumed uniform consumption and negligible axial $P_{{\text{O}}_{2}}$ decline. Subsequent refinements relaxed these assumptions, incorporating axial $P_{{\text{O}}_{2}}$ gradients and variable uptake kinetics, while others highlighted intravascular resistance as a key determinant of oxygen delivery (Goldman, 2008; Popel, 1989). Estimates of minimal $P_{{\text{O}}_{2}}$ thresholds for complete tissue oxygenation across muscles and species have been determined, establishing theoretical ceilings for diffusion-based supply. When applied to the brain and heart, these models reveal conserved geometric rules – radial diffusion limits, consumption-driven $P_{{\text{O}}_{2}}$ gradients and safety margins set by capillary spacing – but scale differently relative to metabolic demand. In the cortex, the larger diffusion radii (~20–25 μm) are sufficient for modest neuronal metabolic rates ($\dot{\text{M}}$), whereas the sinoatrial (SA) node’s denser capillary lattice (~10–15 μm) supports ATP turnover rates ~10-fold higher (Grainger et al., 2021; Ji et al., 2021; Krogh, 1919). This higher demand reduces the diffusion ‘safety margin’ around each cell, even though the same capillary topological principles apply.

In the heart, the ventricular myocardium packs roughly 3000–3500 mm of capillaries into each cubic millimetre, yielding an average inter-capillary distance of ~15 μm, which is narrower than the diameter of a cardiomyocyte (Bassingthwaighte et al., 1974). With each beat, oxygenated blood surges through this evenly spaced lattice, ensuring that every myofilament sits mere micrometres from the source of its next ATP replenishment. With each beat, a surge of oxygenated blood traverses the lattice – predominantly during diastole – so that ATP plus phosphocreatine (PCr) stores (i.e., the combined energy reservoir of the cell) are resupplied before the next contraction.

By contrast, the SA node capillary density averages ~5500–6100 mm/mm^3^, which is about 1.6–2-fold higher than the density of the ventricles. Although denser overall than that of the ventricular myocardium, the capillary network of the mouse SA node is not a perfectly uniform mesh (Grainger et al., 2021; Manning et al., 2025). Grainger et al. (2021) and Manning et al. (2025) showed that vascular density in the murine SA node varies systematically, forming microdomains in which vessel spacing is locally increased. These low-density ‘pockets’ (e.g., ≈880–1000 mm/mm^3^ in the inferior region of the mouse SA node), which coincide with clusters of hyperpolarized pacemaker cells, enhance rhythm generation through a stochastic resonance mechanism by creating sites where subthreshold currents are amplified into reliable APs via noise-assisted threshold crossing. Therefore, rather than a scaled-down copy of the ventricular lattice, the SA node utilizes controlled vascular heterogeneity to fine-tune excitability and sustain robust pacing.

Because stochastic resonance is a relatively new concept in cardiovascular physiology, we briefly explain it here. A stochastic resonance system requires three key elements: (1) a threshold, (2) a sub-threshold periodic oscillator and (3) a noise source. In such systems, the relationship between noise and performance follows a bell-shaped curve: increasing noise levels enhance performance up to an optimal point (resonance), beyond which performance decreases as noise becomes dominant. In biological systems like the SA node, sub-threshold oscillations that would normally fail to trigger APs can be pushed over threshold by an optimal level of biological noise, modulating cellular excitability. These noise effects range from adaptive to pathological depending on the noise magnitude.

Using compartment-targeted ATP sensors in intact nodes and isolated pacemaker cells, Munoz et al. (2025) showed that every heartbeat carries an ATP signature. Specifically, they demonstrated that cytosolic ATP rises transiently with each Ca^2+^ transient and segregates into high-gain and low-gain phenotypes (where gain is defined by the slope of the Ca^2+^–ATP coupling relationship), whereas mitochondrial ATP flux resolves into two stereotyped waveforms: Mode-1 ‘gains’ and Mode-2 ‘dips’. In the intact node, high-gain/Mode-1 phenotypes localize to the superior SA node, and low-gain/Mode-2 phenotypes map to the inferior SA node, mirroring gradients in firing rate, mitochondrial volume and capillary density. Pharmacological analyses place Ca^2+^ upstream of ATP production, with ivabradine (HCN channel antagonist) slowing the ATP cycle without changing amplitude (gating rhythm, not capacity) and thapsigargin (SERCA inhibitor) or carbonyl cyanide 4-(trifluoromethoxy)phenylhydrazone (FCCP; mitochondrial oxidative phosphorylation uncoupler) abolishing beat-locked ATP transients, demonstrating dependence on SR-to-mitochondria Ca^2+^ transfer and intact oxidative phosphorylation. Given decades of work showing that matrix Ca^2+^ entry via the mitochondrial calcium uniporter (MCU) activates Ca^2+^-sensitive dehydrogenases to accelerate OxPhos (Denton & McCormack, 1990; Denton et al., 1980; Kirichok et al., 2004), it is not surprising that SR Ca^2+^ release drives ATP and leads to beat-locked ATP fluctuations; the surprise is that, lacking temporally precise tools, we have been unable to see these transients in intact, beating cells until now.

Three-dimensional (3-D) reconstructions reveal that mitochondrial volume is more than 2-fold greater in superior cells than inferior cells; across pooled SA node myocytes, mitochondrial volume predicts Ca^2+^ load, positioning mitochondria as bandwidth controllers in the hierarchy. In intact tissue, multiphoton imaging using a cytosolic intensity-based ATP-sensing fluorescent reporter (cyto-iATP) shows larger, faster cyto-iATP transients in the superior node (~4 Hz) and more heterogeneous, slower signals in inferior regions (~0.6–2.6 Hz), indicating that ATP production is phase-locked to local firing frequency. Quantitatively, Mode-2 dips reflect faster drawdown of ATP from the matrix than it is produced during the spike, with slower diastolic repayment, consistent with supply-limited microdomains operating nearer the local O_2_ ceiling and favouring low-frequency, fluctuation-rich firing. Pathologically, in an angiotensin II model of early heart failure with preserved ejection fraction (HFpEF) (Manning et al., 2025), selective rarefaction in the superior node precedes bradycardia and heightened beat-to-beat variability, implying that pruning high-gain/Mode-1 territories converts rate-setters into bandwidth-limited followers. In short, capillaries set the ceiling, mitochondria set the bandwidth (ATP production capacity and repayment kinetics) and Ca^2+^ channels set the timing (triggering beat-locked OxPhos). Beat-resolved, compartment-specific support for this proposition is provided in Munoz et al. (2025).

### Network topology and heterogeneity.

Studies on the cerebral cortex reveal a remarkably conserved architecture (Ji et al., 2021). The mean capillary volume fraction is ~2.2% in grey matter, with capillary length density averaging 880–1000 mm/mm^3^ (about 7–3-fold lower than the SA node and ventricles) and intercapillary spacing of ~15 μm in mice (40 μm in humans). Regional heterogeneity reflects functional demands: thalamic regions and the primary somatosensory cortex show elevated densities corresponding to their high metabolic requirements, while hippocampal granular layers demonstrate among the lowest densities at 0.70%. Surprisingly, layer-specific analysis reveals minimal stratification – Layer IV shows only a shallow 15% peak above baseline, contrasting sharply with dramatic neuronal density variations. Yet, as Ji et al. (2021) suggested, this relatively homogeneous mosaic integrates into a small-world mesh whose redundant loops buffer flow when individual segments fail, and whose length density (Σ*L*/*V*) scales inversely with tissue-to-vessel distance such that even 4-fold swings in metabolic demand converge on a common ceiling for tissue $P_{{\text{O}}_{2}}$ (~30 mmHg).

Thus, the cortex achieves metabolic equity not through a uniform supply but rather via a topological safety net that keeps diffusion distances within solvable limits. Functional hyperaemia then provides the rapid overlay, with activity-triggered dilatation initiated in capillaries via K_IR_2.1 and/or TRPA1 channels (Longden et al., 2017; Thakore et al., 2021) – fine-tuned by input from astrocytes and pericytes and specialized segments in the arteriole to capillary transitional (ACT) zone – brightening exactly those vascular pixels that feed an active synapse. Spatial heterogeneity is thus redeemed through temporal agility, a self-adjusting spotlight that follows every burst of spikes without allowing tissue $P_{{\text{O}}_{2}}$ to dip below the floor defined by Ji et al. (2021).

Capillary stalls – brief, localized interruptions of RBC flux – flag points of microvascular fragility, but by themselves do not rewire metabolic capacity. In contrast, capillary rarefaction and loop loss durably lower the oxygen-delivery ceiling and blunt conductive dilatation from the capillary bed to upstream resistance vessels. In the CMIC frame, stalls are acute ‘symptoms’; rarefaction is the chronic ‘disease’ that lowers supply and destabilizes excitability.

Ji et al. (2021) further show that maximum tissue oxygen tension scales because $P_{{\text{O}}_{2},\text{max}}$ is proportional to $L_{D}/\surd \dot{\text{M}}$; thus, the diffusion penalty of capillary loss depends as much on metabolic rate ($\dot{\text{M}}$) as on length density (*L*_D_). In the cortex, where neuronal demand is modest, the lattice can forfeit ~40% of its length before $P_{{\text{O}}_{2}}$ falls from its uniform ~30 mmHg set-point to the hypoxic floor. Shift the same geometry to the SA node – where each pacemaker myocyte consumes ATP at least 10-fold faster – and the tipping point moves left. In this setting, 25–35% rarefaction doubles diffusion distance, halves the $P_{{\text{O}}_{2}}$ reserve and – at the upper end of the range (36%) – slows pacemaking and inflates beat-to-beat variability, as shown by Manning et al. (2025). Female mice, which lose ~50%, overshoot the diffusion limit and teeter on failure.

Expanding on these geometric principles, Grainger et al. (2021) used submicrometre 3-D reconstructions of the murine SA node to quantify vessel density and myocyte-to-vessel proximity. They found that the superior SA node features an interwoven capillary lattice in which the average intercapillary distance is less than 20 μm and nearly 90% of pacemaker cells lie within 10 μm of a perfused microvessel, whereas the inferior region displays sparser networks and mean intercapillary gaps exceeding 35 μm. Such fine-scale heterogeneity provides a structural substrate for regional differences in oxygen delivery and ATP diffusion, reinforcing the idea that the spatial resolution of energetics is set by the capillary mosaic itself.

Topological analyses of the normal SA node vasculature reveal a small-world microvascular network akin to that of the cerebral cortex, differing primarily in scale and density (Grainger et al., 2021; Ji et al., 2021; Manning et al., 2025). In the healthy murine SA node, skeletonized vessel tracings identify ~130–290 closed loops per 500 μm^3^ stack in the superior region *versus* ~44–50 loops in the inferior region. Average branch-to-branch segment length (~15 μm) and branching angles remain conserved across regions and organs, highlighting similar topological rules. Thus, both the heart and brain deploy a redundant loop architecture to buffer perfusion, but the safety margin of SA node capillaries is reduced relative to those in the cortex owing to its lower overall length density and faster ATP turnover. To harmonize comparisons, we report loop density, branch order to feed arteriole and upstream control locus. In the cortex, capillary loops are dense and transmit hyperpolarizing signals to the ACT; in the SA node, loop density is region-dependent (superior > inferior), with upstream feed via SA node arterioles and dense capillary meshes in superior SA node.

In Fig. 1, we illustrate a core principle in the CMIC framework – adapted directly from Grainger et al. (2021), Ji et al. (2021) and Manning et al. (2025) – that capillary networks couple conserved form to flexible function. In Fig. 1A, the fundamental elements of vascular architecture (segment classes, lengths, branching angles, ACT zones) are preserved, providing a loop-rich scaffold. In Fig. 1B, capillary length density scales with local metabolic load – denser meshes under high-demand territories (e.g. superior SA node, ventricular myocardium) than quieter zones – capturing tissue-specific tuning. This tuning, together with redundant loops, yields a common oxygen ceiling: despite regional workload swings, tissue-to-vessel distances and small-world connectivity drive $P_{{\text{O}}_{2}}$ toward similar set points (≈30 mmHg), until rarefaction or upstream constriction erodes redundancy and compromises excitability.

Transport across the endothelium varies by tissue. In the brain, tight junctions restrict paracellular flux, making simple diffusion the primary route for O_2_ and CO_2_, while receptor-mediated transcytosis handles selective macromolecule passage across the blood–brain barrier. In the heart, capillaries are a continuous, non-fenestrated endothelium with tight junctions and abundant caveolae; permeability is tuned more by junctional proteins and flow than by fenestration. We therefore treat cardiac endothelium as diffusion-limited (not leaky), consistent with a high-fidelity coupling between microvascular geometry and oxygen delivery.

### Dynamic regulation and microvascular rarefaction.

Flow through microvascular beds is dynamically regulated by smooth muscle cells (SMCs) and ECs. The myogenic response, a mechanism intrinsic to SMCs that reflects stretch-dependent SMC depolarization (via activation of TRPM4 and other TRP channels), Ca^2+^ entry via L-type Ca_V_1.2 channels and subsequent Ca^2+^-dependent contraction – a process moderated by TRPML1, BK and Kv channel feedback – sets basal tone, while endothelium-dependent vasodilatation (via TRPV4, IK/SK, KIR2.1 and nitric oxide signalling) adjusts perfusion in response to shear stress and metabolic cues (Bagher & Segal, 2011; Gonzales et al., 2014; Ledoux et al., 2008; Longden et al., 2017; Sonkusare et al., 2012; Thakore et al., 2020, 2021).

Microvascular attrition – the pruning or loss of capillaries in ageing, hypertension, diabetes, heart failure and neurodegeneration – injects bioenergetic noise into relationships that are otherwise deterministic (Manning et al., 2024). In the heart, wider capillary spacing desynchronizes neighbouring myocytes, with O_2_ tension and ATP dipping out of phase, APs traversing cells on different metabolic clocks and repolarization dispersion seeding arrhythmias. In the brain, a thinned capillary bed blunts functional hyperaemia, leaving neurons vulnerable to energy crises, spreading depolarizations, and cognitive fatigue. ATP-dependent ion channels and pumps – K_ATP_, Na^+^/K^+^ ATPase and SERCA – translate this energetic jitter into electrical instability, making vascular attrition an active driver of excitability failure.

These observations anchor the first pillar of the CMIC axis: that capillaries dictate how finely and how rapidly O_2_, glucose and other substrates can reach neurons as well as pacemaking myocytes and atrial/ventricular myocytes.

## Mitochondria – translators of flow into function

If capillaries dictate where metabolic substrates are delivered, mitochondria determine when those substrates are converted into ATP. Strategically positioned – either locked in crystalline arrays (heart) or trafficked like courier drones (neurons) – mitochondria transform blood-borne fuel into the rapid energy pulses that maintain ionic gradients and sustain channel activity. Within the CMIC axis, mitochondria serve as temporal transformers, converting vascular flow into rhythmic or adaptive ATP signals.

Mitochondrial organization differs substantially between cardiac and neural tissues (Fig. 2). In neurons, mitochondria are distributed throughout the soma and exhibit dynamic trafficking along microtubule networks, accumulating at synapses and nodes of Ranvier in response to local metabolic demands (Fig. 2A). Within these specialized neuronal compartments, mitochondria support synaptic vesicle cycling and maintain ion homeostasis at sites of peak energy consumption. In cardiomyocytes, mitochondria demonstrate a highly ordered arrangement, positioned in longitudinal arrays between myofibrils and adjacent to the SR (Fig. 2B). This structured organization minimizes diffusion distances for Ca^2+^ ions released during excitation–contraction coupling, facilitating tight temporal coordination between mitochondrial ATP production and contractile energy demands (Bertero & Maack, 2018).

Live biosensor imaging reveals just how rhythmic this translation can be in the heart. Pacemaking and ventricular myocytes display millisecond-scale oscillations, exhibiting a gain phase during systole as mitochondrial output surges, followed by a dip that bottoms out moments before the next depolarization (Munoz et al., 2025; Rhana et al., 2024, 2025). These gain–dip domains – only a sarcomere wide – vanish if Ca^2+^ uptake into mitochondria falters or SR–mitochondrial tethers are severed. K_ATP_ channels open transiently during the dip, subtly abbreviating APs and reshaping repolarization reserve. Thus, the disappearance of gain–dip domains leaves a metabolic stutter that bleeds into electrophysiology.

Unlike cardiomyocytes, neurons do not march to a metronome. Bursts of synaptic activity evoke phasic mitochondrial Ca^2+^ spikes, accelerating NADH oxidation and doubling perisynaptic ATP within hundreds of milliseconds. Direct evidence supporting the idea that axonal excitability lives on an equally thin ATP margin comes from real-time imaging in the optic nerve. In the study by Trevisiol et al. (2017), the FRET-based sensor ATeam1.03 was expressed in Thy1-positive neurons and paired with ratiometric confocal imaging and compound AP recordings. During high-frequency stimulation, compound AP frequency fell in lock-step with a rapid drop in axonal ATP, showing that electrical failure coincides with local energy bankruptcy. Crucially, blocking lactate utilization broke this correlation, indicating that oligodendrocyte-derived lactate – not axonal glycolysis alone – sustains mitochondrial ATP production in active white-matter tracts. These findings extend the CMIC framework by suggesting that a glial relay is needed for capillary-fed substrates to reach fast-firing axons.

Figure 3 links Ca^2+^ signalling to mitochondrial ATP output in two excitable systems. In neurons (Fig. 3A), the upper frame shows lower Ca^2+^ influx with weak endoplasmic reticulum (ER)–mitochondria coupling: minimal mitochondrial Ca^2+^ uptake yields small, delayed ATP production, favouring K_ATP_ channel opening and indirectly weakening excitability by modulating SERCA and Na^+^/K-ATPase activity. The lower frame in the figure shows higher Ca^2+^ influx with tighter ER–mitochondria coupling. Robust mitochondrial Ca^2+^ entry accelerates and amplifies ATP synthesis, closing K_ATP_ and sustaining pumps to preserve gradients and firing reliability. Cardiac myocytes (Fig. 3B) mirror this logic: with low Ca^2+^ influx (upper), limited SR–mitochondria crosstalk generates modest ATP, predisposing to K_ATP_ activation and reduced SERCA/Na^+^/K^+^-ATPase reserve; with high Ca^2+^ influx (lower), efficient SR-to-mitochondria Ca^2+^ transfer drives rapid, beat-locked ATP surges that suppress K_ATP_ and maintain pump-supported excitability. Viewed through the CMIC lens, mitochondria translate capillary-supplied substrates into a temporal code for excitability: (1) capillaries set the spatial resolution of substrate delivery; (2) mitochondria convert this input into ATP waveforms – rhythmic in the heart, adaptive in the brain; and (3) with few exceptions (e.g. K_ATP_, cell-surface P2X receptors), ion channels do not sense ATP directly. Rather, channel activity is governed primarily by ATP-dependent maintenance of ionic gradients and phosphorylation state, which deteriorate as ATP availability wanes.

## Ion channels – metabolic integrators at the membrane

Ion channels function beyond mere ionic conductance, serving as metabolic sensors that transduce fluctuations in local ATP availability into corresponding electrical signals (Hille, 2001). As the terminal effector of the CMIC axis, their activity provides real-time readout of capillary perfusion and mitochondrial function. Under conditions of adequate ATP delivery, cellular excitability maintains precise temporal and spatial coordination; conversely, when substrate supply becomes compromised, channels introduce bioenergetially derived variability into electrical signalling – a response that initially serves an adaptive function but may progress to pathological states including arrhythmia, seizure or contractile dysfunction under conditions of sustained metabolic insufficiency.

Certain channel populations demonstrate direct nucleotide sensitivity through molecular binding interactions. The prototypical K_ATP_ channel complex remains closed during metabolic sufficiency, thereby preserving membrane excitability, and undergoes conformational opening upon ATP depletion, resulting in AP abbreviation and consequent reduction in cellular energy expenditure (Inagaki et al., 1995; Nichols, 2006; Noma, 1983).

A parallel layer of metabolic integration is supplied by electrogenic pumps. The Na^+^/K^+^-ATPase and SERCA set ionic gradients and Ca^2+^ cycling, and their rates scale with ATP availability. The Na^+^/K^+^-ATPase, responsible for roughly 40% of neuronal (Attwell & Laughlin, 2001; Howarth et al., 2012) and 20% of cardiac (Aksentijevic et al., 2020; Matsuoka et al., 2004) ATP expenditure, generates a hyperpolarizing leak current whose magnitude scales with ATP availability, whereas SERCA sets the pace of Ca^2+^ waves, and thereby the workload of mitochondria themselves, by pulling Ca^2+^ back into the SR/ER. When ATP wanes, both pumps slow, ion gradients sag and the membrane potential drifts toward instability.

Yet another layer rests on phosphorylation-dependent modulation. Voltage-gated Na^+^ and Ca^2+^ channels, inward-rectifier K^+^ channels, and many ligand-gated pores rely on protein kinase A (PKA), protein kinase C (PKC) or Ca^2+^/calmodulin-dependent kinase II (CaMKII) to maintain optimal gating kinetics (Hille, 2001). Every phosphorylation event costs one molecule of ATP, so when local pools shrink, kinase activity lags, phosphorylation states decay and channel kinetics slide, leading to slower inactivation of voltage-dependent Na^+^ (Na_V_) channels in the heart and diminished facilitation of voltage-dependent Ca^2+^ (Ca_V_) channels in neurons – subtle shifts that erode the safety margin for electrical stability.

Purinergic signalling uses ATP as an extracellular cue via P2X (ionotropic) and P2Y (metabotropic) receptors to coordinate vascular tone, neurotransmission and immune responses – linking energy status to excitability through a paracrine route (Burnstock, 2007).

In metabolically stressed tissues, using ATP for communication may appear paradoxical. However, this apparent inefficiency may be outweighed by the functional necessity of rapid, localized communication. In this view, ATP release is analogous to investing in a robust communication network – resource-intensive in the short term, but essential for maintaining coordinated function and survival in complex cellular environments. In tissues where precise coordination between cell types is required, such as the neurovascular unit, the benefits of timely and reliable signal transmission may far exceed the energetic costs.

These ATP-sensitive mechanisms collectively transform metabolic variability into electrical variability. Minute-to-minute fluctuations in ATP provoke K_ATP_ channel openings, Na^+^/K^+^-ATPase lag and SERCA latency. In the myocardium, the resulting dispersion of AP duration seeds re-entrant circuits; in the cerebral cortex, such heterogeneity could bias neurons toward hyper-excitability. Crucially, such dysfunction need not stem from genetic channelopathy; it can originate upstream, in microvascular rarefaction or mitochondrial uncoupling – points of intervention that are, at least in principle, modifiable.

Phosphatidylinositol 4,5-bisphosphate (PIP_2_), a minor but essential membrane phospholipid, is another central currency linking cellular energetics and ion channel function (Earley & Kleinfeld, 2021). PIP_2_ directly modulates the activity of numerous ion channels, including K_IR_2.1, K_ATP_ and TRPM4 (Harraz et al., 2020; Suh & Hille, 2008). At the plasma membrane (PM), phosphatidylinositol (PI) is sequentially phosphorylated to PIP_2_ by two Mg-ATP-dependent kinases: PI4KIII*α* (converting PI to PI4P) and PIP5K (converting PI4P to PIP_2_) (van den Bout & Divecha, 2009). Because these lipid kinases function at ER–PM junctions, where lipid pools are small and functionally compartmentalized, their activity depends on local ATP microdomains, which are preferentially maintained by membrane-proximal glycolysis. Moreover, although ATP depletion also threatens the electrochemical gradient, ionic pumps such as the Na^+^/K^+^-ATPase have a higher affinity for ATP (*K*_m_ ~150 μM) (Friedrich et al., 1996) than PI4KIII*α* (*K*_m_ ~700 μM) (Balla & Balla, 2006), making PIP_2_ synthesis more sensitive to energetic compromise than membrane potential maintenance. As a result, even modest decreases in local ATP can cause a rapid decline in PIP_2_ levels at the plasma membrane. Thus, loss of PIP_2_ and consequent ion channel dysfunction may be an early and sensitive marker of declining cellular energy status.

Experimental support for this concept comes from two studies by the Nelson laboratory. In mouse models of CADASIL (cerebral autosomal dominant arteriopathy with subcortical infarcts and leukoencephalopathy), the most common genetic cerebral small vessel disease (cSVD) (Dabertrand et al., 2021), and 5xFAD familial Alzheimer’s disease (Mughal et al., 2021), ATP depletion leads to PIP_2_ depletion and loss of K_IR_2.1 channel activity in brain capillary ECs, and impaired capillary-to-arteriole dilatation and functional hyperaemia. In contrast, work in a Gould syndrome cSVD model demonstrated that PIP_2_ depletion, and associated loss of K_IR_2.1 activity in ECs and SMCs (Thakore et al., 2023), as well as TRPM4 inactivity in SMCs, occurs independently of ATP decline and is instead driven by elevated PI3K activity (Yamasaki et al., 2023). These defects were shown to impair pressure autoregulation, capillary-to-arteriole dilatation and functional hyperaemia in the brain. Thus, PIP_2_ depletion is a rapid, ATP-sensitive trigger of neurovascular signalling failure that occurs well before catastrophic loss of membrane potential. Because PIP_2_ controls multiple ion channels essential for brain blood-flow regulation, its loss, whether from metabolic insufficiency or pathological kinase activation, represents a convergent and druggable choke point in the cascade leading from vascular dysfunction to cognitive decline (Hachinski, 1988).

Within the CMIC axis, then, ion channels serve as real-time reporters of metabolic integrity. A flat, noise-free membrane potential signals seamless capillary delivery and mitochondrial translation, whereas a jittery potential heralds a breach in the energy supply chain.

## The brain’s adaptive energy supply – neurovascular coupling and functional hyperaemia

Neurons operate under strict metabolic constraints. Due to their minimal ATP storage capacity, neuronal function requires continuous delivery of oxygen and glucose. Cerebral function is therefore contingent upon vascular regulatory mechanisms that redistribute blood flow to regions of increased synaptic activity within seconds. The neurovascular coupling mechanisms that coordinate neuronal activity with selective augmentation of regional perfusion generate functional hyperaemic responses – localized increases in capillary and arteriolar flow – that replenish ATP prior to depletion of intracellular reserves below critical operational thresholds.

The brain’s dense capillary network functions as a distributed sensor array, detecting local neural activity and translating it into propagating electrical and Ca^2+^ signals that promote upstream arteriolar dilatation, thereby matching blood flow with metabolic demand. Capillary ECs express multiple, highly sensitive membrane receptors and ion channels. For example, elevations of perivascular K^+^, released during synaptic activity, activate K_IR_2.1 channels, producing a membrane hyperpolarization that conducts rapidly along the endothelium (Longden et al, 2017) via Cx37/Cx40 gap junctions (Krolak et al., 2025) toward the arteriole, closing L-type Ca^2+^ channels on SMCs, reducing tone and thereby promoting dilatation. In parallel, ROS-derived lipid peroxidation products activate TRPA1 channels in capillary ECs, initiating intercellular Ca^2+^ waves that are propagated by pannexin 1-mediated ATP release and P2X receptor activation (Thakore et al., 2021). These Ca^2+^ signals are converted to hyperpolarizing electrical signals by small- and intermediate-conductance Ca^2+^-activated K^+^ channels in the ACT zone. Additionally, activation of prostaglandin E_2_ (EP) receptors on capillary ECs can elicit conducted dilatation of upstream parenchymal arterioles (Rosehart et al., 2021). The integration of these diverse receptor-driven signals by the capillary network orchestrates the timing, magnitude and spatial distribution of functional hyperaemic responses.

While ECs ‘light the fuse’, astrocytes and pericytes regulate the capillary radius and timing. Astrocytic endfeet ensheathe arterioles and modulate Ca^2+^ waves, which fine-tune dilatation (Diaz-Castro et al., 2023; Gordon et al., 2008), whereas pericytes enwrapping capillaries either constrict or relax segments to steer RBC flux toward the most active synapses (Longden & Isaacs, 2025). This tri-cellular choreography ensures that the hyperaemic response is not a flooding event but rather a spatially precise ‘spotlight’, brightening exactly where synaptic ATP demand will peak. Lesion studies further provide support for the idea of a ‘conductive’ capillary network that normally propagates dilatory signals upstream to arterioles. In the mouse cortex, two-photon microlesions that cause capillary regression were found to trigger constriction of vascular segments upstream of the lesion, blunting vasomotor dynamics and reducing flow, not only at the lesion but across otherwise intact downstream capillaries (Bonney et al., 2024). The chokepoint in this dynamic is the ensheathing pericyte-enwrapped ACT zone, which exhibits chronic diameter reduction after regression events, functionally lowering the local O_2_ ceiling and decoupling conducted dilatation from neural demand (Bonney et al., 2024). Thus, when nodes are lost, the conduction path fails and supply to broader territories falls (Bonney et al., 2024). This bottleneck logic predicts a variability-first phenotype in axonal conduction that mirrors activity-locked ATP vulnerabilities observed in white matter (Trevisiol et al., 2017).

Deep multiphoton imaging reveals sparse, wide–reaching principal cortical venules that selectively drain deep tissues (layer 6 and corpus callosum). With ageing, capillaries in deep principal cortical venule tributaries selectively constrict and rarefy, producing mild but persistent hypoperfusion coupled with microgliosis, astrogliosis and demyelination; this induces a comparable flow drop via carotid stenosis recapitulating the layer 6/callosal pathology (Stamenkovic et al., 2025). Consistent with white-matter ATP imaging results (Trevisiol et al., 2017), even mild, chronic hypoperfusion should prolong ATP repayment and depress compound APs prior to evidence of overt tissue loss.

The Stamenkovic et al. (2025) study pinpoints capillary diameter as the dominant determinant of local flow in these deep beds, highlights their vulnerability to regression and pericyte loss, and identifies principal cortical venules as drainage bottlenecks, showing that small calibre changes in deep tributaries translate into system–level impedance of outflow and sustained supply side under–perfusion (Stamenkovic et al., 2025). In CMIC terms, venous architecture can impose a drainage–limited ceiling on perfusion that holds mitochondrial bandwidth hostage in ATP–intensive white–matter interfaces.

The physiological efficacy of functional hyperaemia relies on its feed-forward nature; blood flow increases milliseconds before intracellular ATP reserves are depleted, preventing a ‘running-on-fumes’ scenario and preserving spike fidelity. However, disruption of this capillary-level control, whether via rarefaction or pericyte dysfunction, rapidly blunts the response. The resulting localized energy deficits stall the Na^+^/K^+^-ATPase, biasing neurons toward depolarization block or runaway excitation. These micro-ischaemic events create fertile ground for spreading depolarizations and seizures (Dreier, 2011), as well as the synaptic failures underlying cognitive fatigue and neurodegeneration (Iadecola, 2017). In the context of epilepsy, this coupling can become maladaptive; primary research demonstrates that severe seizures can induce a paradoxically severe vasoconstriction and local hypoxia – despite high metabolic demand – which drives the post-ictal state and perpetuates neuronal dysfunction (Farrell et al., 2016). In the CMIC view, neurovascular coupling represents the capillary covenant linking energy and thought – the precise locus where metabolism meets mind.

## The heart’s rhythmic supply chain – coupled metabolism and contraction

Each cardiac cycle represents a critical metabolic challenge (Rhana et al., 2024, 2025). Within approximately 300 ms, cardiomyocytes must execute depolarization, calcium influx, contraction, relaxation and ionic homeostatic restoration – a sequence requiring ATP consumption of such magnitude that any temporal dissociation between utilization and synthesis results in immediate electrical dysfunction. Recent live-cell imaging studies have provided empirical validation of long-standing theoretical predictions: mitochondrial ATP production exhibits beat-to-beat oscillations synchronized with APs. Utilizing genetically encoded biosensors, Rhana et al. (2024, 2025) documented millisecond-resolution ATP fluctuations characterized by peaks during early systole and nadirs immediately preceding subsequent depolarization. Subsequent investigations by Manning et al. (2025) demonstrated spatial localization of these oscillations to sub-sarcomeric domains, identifying alternating zones of ATP enrichment and depletion within microdomains spanning only several micrometres. These findings indicate that mitochondria generate energy in discrete temporal packets precisely synchronized to contractile cycles, effectively transducing coronary perfusion into cyclical ATP provision aligned with each cardiac contraction.

This beat-to-beat generation and consumption of ATP leaves energetic reserves low. By late diastole, many myocytes hover perilously close to the K_ATP_ activation threshold – ‘paycheck-to-paycheck’ energetics where the next ATP deposit must ‘clear’ before the next AP causes an ‘overdraft’. When coronary perfusion is healthy and the capillary lattice is intact, this choreography is flawless – capillaries fill during diastolic relaxation, mitochondria spike ATP during systole and ion channels ride a flat metabolic baseline.

Trouble begins with vascular rarefaction (Manning et al., 2024, 2025). As capillary density falls, whether from hypertension, diabetes or ageing, the spatial uniformity of oxygen delivery disintegrates. Some sarcomeres receive their ATP supply on time, whereas adjacent sarcomeres experience a delay. The ensuing metabolic asynchrony fractures the tight coupling between Ca^2+^ handling and ATP supply, slowing SERCA, causing cytosolic Ca^2+^ to linger, stretching repolarization and widening the dispersion of AP duration. On a macro-scale, the tissue becomes an arrhythmic patchwork primed for re-entry. Experimental evidence confirms that these metabolic heterogeneities are arrhythmogenic; for example, local mitochondrial depolarization caused by oxidative stress creates a ‘metabolic sink’ that destabilizes the electrical AP, directly precipitating re-entrant ventricular tachycardia (Akar et al., 2005). What appears electrophysiological at the bedside is, in CMIC terms, a supply-chain failure at the capillary link.

Thus, the heart exemplifies the CMIC axis in its most time-compressed form: capillaries deliver, mitochondria translate and ion channels fire – all within a single cardiac cycle. Break the rhythm anywhere and the entire supply chain falters, transforming ordered contraction into the electrical chaos of arrhythmia.

## Broader implications of the unified model and experimental frontiers

Figure 4 crystallizes the CMIC logic under two operating regimes. In Fig. 4A (low-demand/limited coupling), a relatively sparse capillary bed feeds a modest mitochondrial network. Weak SR/ER-to-mitochondria Ca^2+^ transfer blunts and delays ATP synthesis, so local ATP falls toward the end of activity bouts. In this state, ATP-dependent electrogenic work is fragile: K_ATP_ channels tend to open, while SERCA and the Na^+^/K^+^-ATPase lose reserve – together depressing excitability, pacing reliability and contraction. In Fig. 4B (high-demand/strong coupling), a dense microvascular lattice sustains a dense mitochondrial reticulum. Tight SR–mitochondrial Ca^2+^ coupling accelerates and amplifies ATP production, generating rapid, appropriately timed ATP surges that close K_ATP_, power SERCA-mediated Ca^2+^ store refilling and maintain Na^+^/K^+^-ATPase flux – thereby stabilizing high-frequency excitability, pacemaking and contractile performance.

Viewed through the framework of Ji et al. (2021) and Grainger et al. (2021), the capillary topology underlying the cartoons in Fig. 4A and B is conserved (loop-rich, small-world), while density scales with local metabolic load; mitochondrial abundance and Ca^2+^-control bandwidth scale in parallel. This graded scaling allows very different workloads to converge on similar tissue $P_{{\text{O}}_{2}}$ ceilings, while mitochondria translate perfusion into tissue-specific ATP waveforms – rhythmic in heart, adaptive in brain. Thus, the CMIC axis unifies form and function: (1) capillaries set the spatial resolution and ceiling of substrate delivery; (2) mitochondria convert that input into temporally coded ATP; and (3) with few exceptions (e.g. K_ATP_, P2X receptors), ion channels read ATP indirectly via pump-maintained gradients and phosphorylation state, both of which deteriorate as ATP wanes. Disruptions at any CMIC link – rarefaction, reduced mitochondrial bandwidth or channel/pump insensitivity to energetic status – produce bioenergetic shortfalls that present as arrhythmias, heart-failure phenotypes, cognitive fatigue or seizure susceptibility.

Experimental frontiers follow directly: map ATP microdomains against capillary density and topology; define perfusion–excitability thresholds by titrating O_2_ during paced/optogenetic activity; tune mitochondrial Ca^2+^ uptake to shift ATP timing and amplitude; and quantify how K_ATP_, SERCA and Na^+^/K^+^-ATPase act as convergent effectors that couple energetic status to excitability across the A→B spectrum.

Experimentally engaging the CMIC framework demands new tools. First is *in vivo* ATP imaging with sufficient spatiotemporal resolution to capture a cardiomyocyte’s beat-locked dip or a dendrite’s burst-driven surge. Genetically encoded reporters paired with advanced optics and deep-tissue microscopy are beginning to deliver this, but multispectral, organ-scale maps remain elusive. Parallel progress must come from CMIC-aware computational models. Existing cardiac and cortical simulations excel at voltage propagation yet treat ATP as a constant. Next-generation models should hard-wire vascular topology, mitochondrial kinetics and channel gating into a single dynamical framework, enabling predictions of how a missing capillary branch ripples outward to AP dispersion or synaptic failure.

Notably, the CMIC framework offers a mechanistic lens on why AAV1-mediated SERCA2a gene therapy has so far failed to translate. In the CUPID-2 trial, an intracoronary infusion of AAV1/SERCA2a did not reduce recurrent heart-failure events or mortality compared with placebo, despite encouraging signals in earlier phase studies (Greenberg et al., 2016). Subsequent analyses from the SERCA-LVAD study and related programmes indicate that myocardial AAV1/SERCA2a vector DNA and SERCA2a transgene expression were generally low or undetectable in human hearts, implying that target engagement was far below that achieved in preclinical models (Hulot et al., 2016; Lyon et al., 2020). From a CMIC perspective, both inadequate local gene transfer and the energetic context matter: in ventricles with coronary microvascular rarefaction, impaired coronary flow reserve and disrupted SR–mitochondrial coupling – features increasingly recognized in advanced heart failure – upregulating an ATP-hungry pump such as SERCA2a risks further straining a limited ATP budget. Thus, the CMIC lens suggests that durable benefit will probably require strategies that both improve the upstream energetic supply chain (e.g. enhancing capillary density and microvascular function, restoring mitochondrial–SR tethering or augmenting capillary-initiated signalling) and achieve adequate local SERCA2a expression, rather than modulating downstream ATPases in isolation.

In sum, the CMIC model suggests that restoring electrical stability requires rebuilding the systems that sustain it – not only modulating channels in isolation but also strengthening the upstream links that feed them: stimulating angiogenesis to quell arrhythmias, restoring capillary K^+^-channel signalling to temper seizures and mitigate cognitive decline, and re-coupling mitochondria to the SR to avert heart failure. The lesson is simple and profound: excitability is ecosystem-dependent and rooted in metabolic synergy.

## Supporting information

Additional supporting information can be found online in the Supporting Information section at the end of the HTML view of the article. Supporting information files available:

Peer Review History

## Figures and Tables

**Figure 1.**
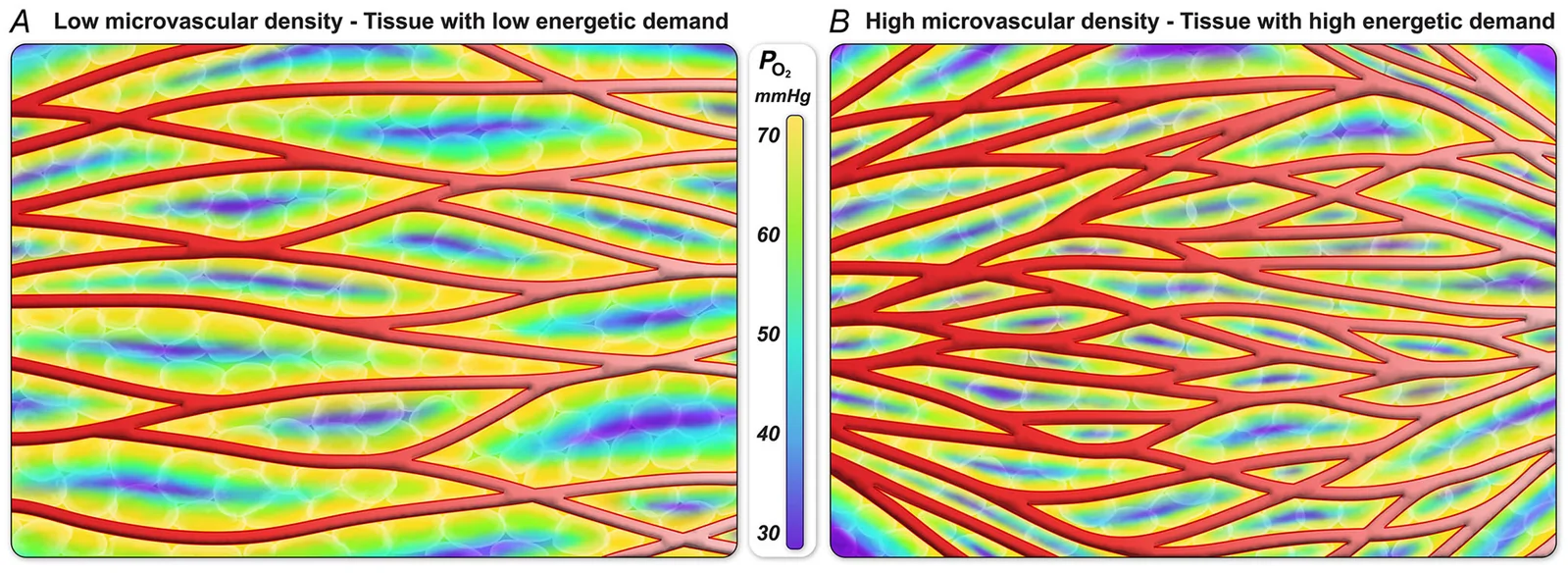
Capillary density sets the oxygen ceiling *A*, low microvascular density in tissue with modest energetic demand. Widely spaced capillaries (red) create long diffusion paths and shallow $P_{{\text{O}}_{2}}$ fields (yellow heatmap), lowering the spatial resolution of oxygen delivery and limiting peak aerobic throughput. *B*, high microvascular density in tissue with high energetic demand. Tighter capillary spacing produces overlapping $P_{{\text{O}}_{2}}$ fields and a higher local oxygen ceiling. The vertical bar indicates relative $P_{{\text{O}}_{2}}$ (top, high; bottom, low). This panel illustrates the first tier of the CMIC framework: capillary architecture sets the maximum sustainable oxidative supply.

**Figure 2.**
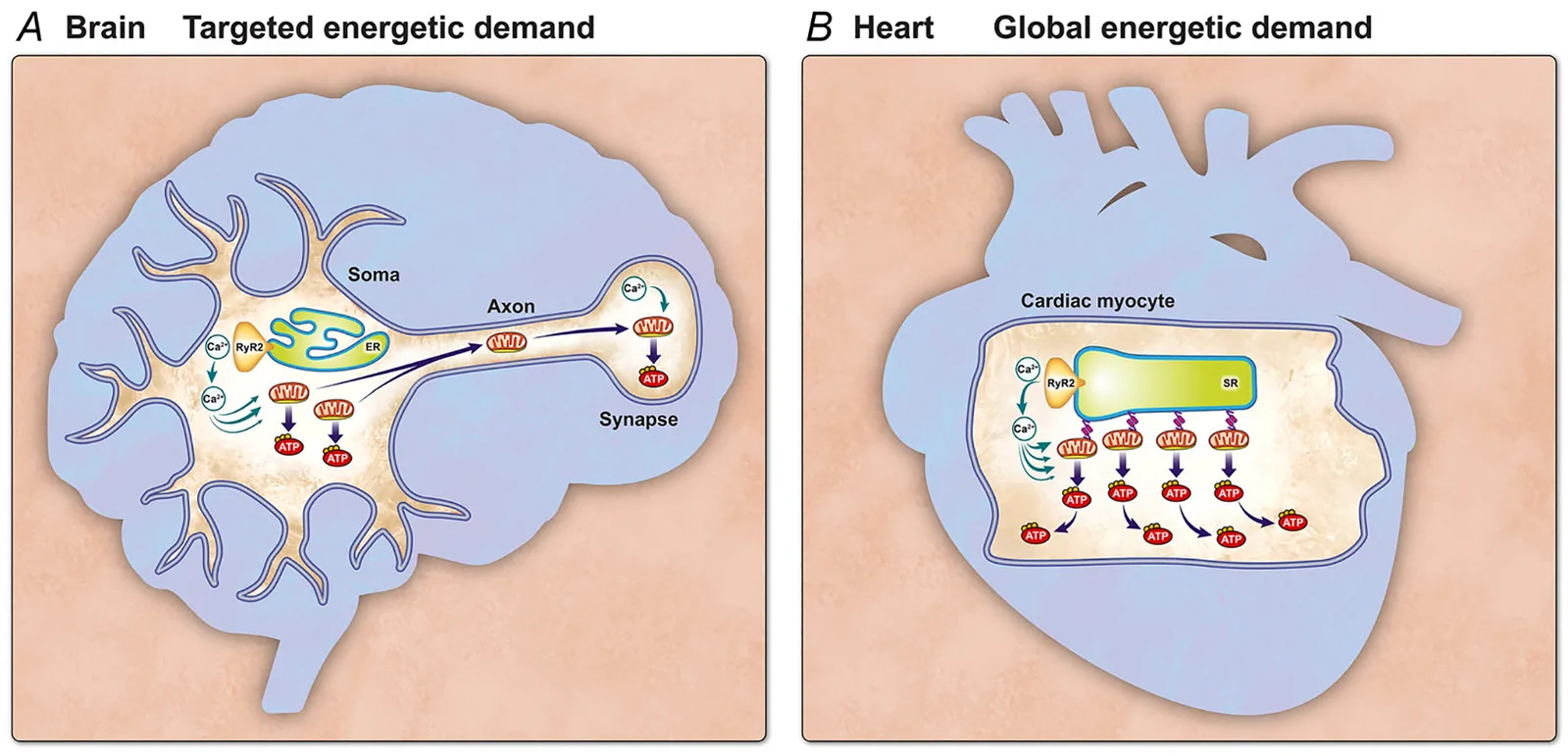
Matching network topology to demand in the brain and heart *A*, brain (lower average energetic demand). In neurons, Ca^2+^ released from ER (RyR2) and modest Ca^2+^ entry support localized mitochondrial ATP production near synapses; mitochondria are transported along axons to specific sites, including synapses. *B*, heart (higher energetic demand). In ventricular myocytes, mitochondria are arrayed beneath the sarcoplasmic reticulum (SR) and T-tubules so that beat-to-beat Ca^2+^ signals drive oxidative phosphorylation in many parallel microdomains, providing high-bandwidth ATP delivery for ion transport and contraction. Together, these schematics emphasize how tissue-specific organization aligns energy supply with workload.

**Figure 3.**
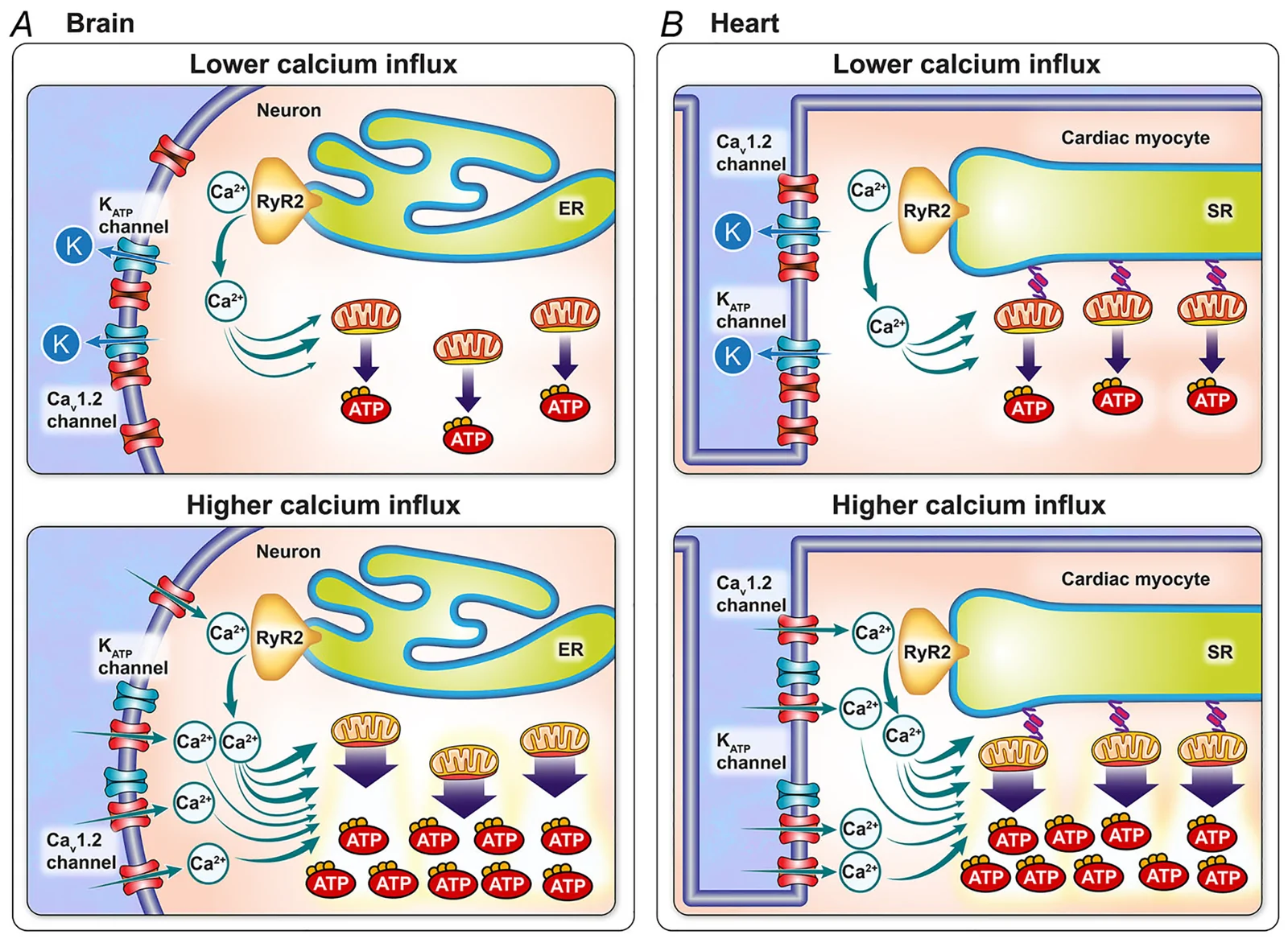
Ca^2+^ influx sets the timing of ATP production Left, brain; right, heart. For each tissue, upper panels depict lower Ca^2+^ influx and lower panels depict higher Ca^2+^ influx. Ca_V_1.2-mediated Ca^2+^ entry and ER/SR Ca^2+^ release (RyR2) generate local Ca^2+^ microdomains that stimulate nearby mitochondria to produce ATP (red organelles; ATP icons). As Ca^2+^ influx increases, mitochondrial ATP output scales, supporting greater transport work; when ATP is ample, K_ATP_ channels remain closed, stabilizing excitability. This figure illustrates the third tier of the CMIC framework: Ca^2+^ channels set the temporal gating of oxidative phosphorylation and ATP availability.

**Figure 4.**
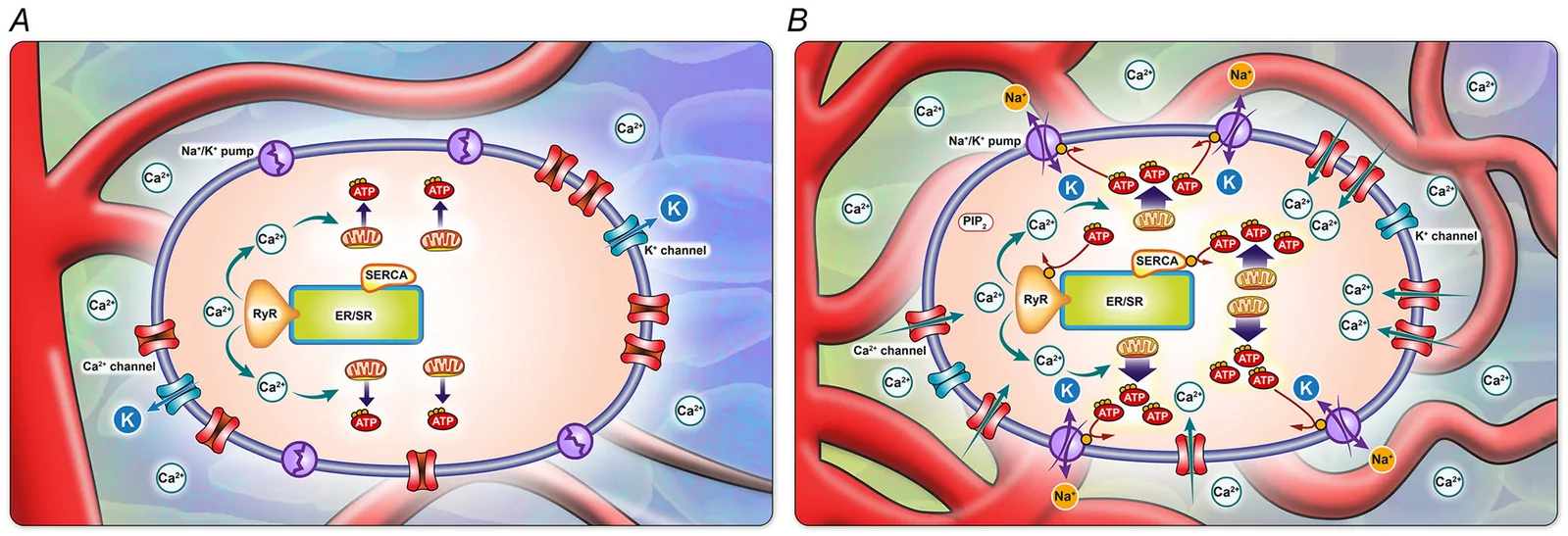
Integrated electro-metabolic coupling at low *vs*. high workload *A*, balanced state (lower workload/adequate supply). Moderate Ca^2+^ influx through Ca_V_ channels, controlled SR cycling (RyR/SERCA) and Na^+^ influx matched by Na^+^/K^+^-ATPase are supported by mitochondrial ATP microdomains. K^+^ channels maintain repolarization; extracellular $P_{{\text{O}}_{2}}$ is sufficient. *B*, high-demand or supply-limited state. Enhanced Ca^2+^ entry (multiple Ca^2+^ routes), stronger SR cycling and greater Na^+^ load increase ATP demand. Mitochondria upshift ATP production, but if demand approaches the vascular/oxygen ceiling, local ATP dips can emerge, taxing the Na^+^/K^+^ pump and favouring opening of ATP-sensitive pathways (e.g. K_ATP_), while PIP_2/s_-messenger signalling and TRPM4 further influence excitability. This synthesis highlights CMIC hierarchy under stress: capillary supply sets the ceiling, mitochondria provide bandwidth and Ca^2+^ influx sets timing – together determining whether excitability is maintained or destabilized.
